# Current Standards in the Management of Cerebral Metastases

**DOI:** 10.1155/2012/493426

**Published:** 2011-11-10

**Authors:** Pablo Goetz, Julius O. Ebinu, David Roberge, Gelareh Zadeh

**Affiliations:** ^1^Division of Neurosurgery, Toronto Western Hospital, 399 Bathurst, Toronto, ON, Canada M5T 2S8; ^2^Département de Radio-Oncologie, Hôpital Notre Dame, Université de Montréal, Montréal, QC, Canada H3C 3J7

## Abstract

The last 30 years have seen major changes in attitude toward patients with cerebral metastases. This paper aims to outline the major landmarks in this transition and the therapeutic strategies currently used. The controversies surrounding control of brain disease are discussed, and two emerging management trends are reviewed: tumor bed radiosurgery and salvage radiation.

## 1. Introduction

### 1.1. Background

Brain metastases are the most common form of brain cancer and exceed the number of primary brain tumors by at least fourfold, with an estimated 98,000–170,000 new cases of brain metastases per year in North America [1, 2]. These figures will further rise as a result of an ageing population, increasing use of improved diagnostic imaging and advancements in systemic and local cancer therapies. The primary tumors most likely to metastasize to the brain are located in the lung (50%), breast (15–25%), skin (melanoma) (5–20%), colon-rectum, and kidney [3, 4]. In addition, most other malignant tumors can metastasize to the brain. In up to 15% of patients, the primary site is unknown. 

The diagnosis of brain metastases (BM) is devastating for both patients and caregivers as evidenced by the heavy burden of disease and significant impact on quality of life (QOL). Clinical manifestations can include focal neurological deficits, seizures, raised intracranial pressure, and alteration in cognition, personality, and ultimately functional status. Untreated, patients with BM will typically live less than two months [5]. 

Patients harbouring BM present a therapeutic challenge to clinicians. This is primarily due to the heterogeneity in cancer type and extent, patient demographics and clinical status, and treatment history and options. To better understand the factors influencing survival, a recursive partitioning analysis (RPA) of data from three Radiation Therapy Oncology Group (RTOG) prospective brain metastases trials was performed identifying 3 classes of patients based on age, performance status, systemic disease control, and presence of extracranial metastases (Table 1) [6]. These classes predict median survival (7.1, 4.2, and 2.3 months for classes I, II, and III patients, resp.) and suggest that many RPA III patients will benefit little from aggressive treatment. More aggressive strategies are recommended in younger patients with controlled systemic cancer, minimal extra-cranial metastases, and high-performance status.

Based on a review of more than four thousand BM patients, the disease-specific graded prognostic assessment (DS-GPA) can produce survival estimates better tailored to the primary malignancy [7]. In some cancers, additional factors will be of prognostic importance (hormone receptor status for breast cancer and number of brain metastases in lung cancer, melanoma, and renal cell carcinoma) while in others performance status will dominate prognosis (gastrointestinal malignancies) [8].

The ongoing expansion in the number and complexity of therapeutic options seen over the past three decades has added further intricacy to the management of BM patients. Treatment decisions must therefore be tailored to each individual, based on a complex array of patient- and tumour-specific characteristics, as no single algorithm is appropriate for every patient. Close collaboration is required between general medical and radiation oncologists, neuro-oncologists, and neurosurgeons in a multidisciplinary setting where multiple treatment modalities can be offered. 

Finally, there is a lack of consensus amongst practitioners on the optimum treatment strategy and it is often the experience of the local treating team that governs decision making. This trend is underpinned by persistent controversies and limitations in the data supporting management guidelines. Whilst accepted medical practice, many of the treatment regimes prescribed are not founded on rigorous prospective data. More evidence-based driven treatment paradigms are required. This review aims to discuss the existing literature, some of the key on-going controversies, and emerging trends surrounding local control (LC) of BM.

## 2. Therapeutic Strategies and Supporting Evidence

**Figure 1:**
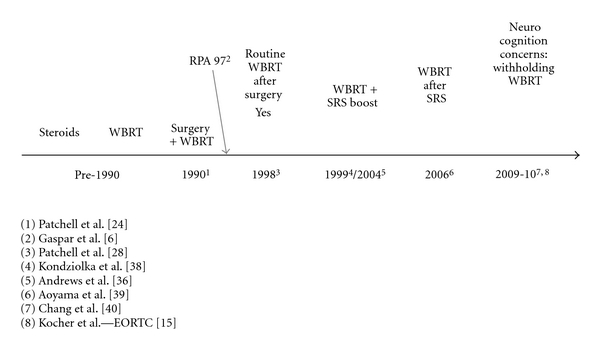
Timeline showing some of the landmarks in the management of brain metastases and related publications.

The last 30 years have seen important changes in the management of brain metastases. Below is a timeline displaying the major landmarks and some of the notable related publications (Figure 1).

### 2.1. Corticosteroids

The palliative use of corticosteroids in patients harbouring cerebral metastases is necessary for symptom control as it aids in alleviating peritumoral edema [9, 10]. However, the dose and duration of corticosteroid use is variable. Although recommended for short-term symptom control, patients often remain on prolonged regimens with related complications. These complications are well known and can negatively impact quality of life due to the associated adverse effects such as myopathy, hyperglycemia, weight gain, immune suppression, insomnia, emotional lability, and occasional severe psychiatric disturbances.

### 2.2. Whole-Brain Radiation Therapy

Although only studied in a 48-patient trial from the pre-CT scan era, whole-brain radiation therapy (WBRT) has been the mainstay of metastatic brain tumour therapy for decades. Following WBRT, overall survival is typically 3-4 months. The goals of WBRT include relief of symptoms from existing BM and prevention of future BM. Common North American regimens use parallel-opposed megavoltage beams to deliver 20–37.5 Gy in 5–15 daily fractions over 1–3 weeks. Acute complications include encephalopathy, cerebral edema, nausea and vomiting, alopecia, dermatitis, mucositis, otitis externa, and fatigue. Late complications can include optic and otic toxicities, endocrinopathies, and decline in neurocognitive function. Although potentially debilitating, the real incidence of severe radiation encephalopathy is poorly documented [11]. In a study published in 1989, a dementia rate of 11% was reported in a series of 47 patients. Of particular note, four of these patients received a high dose per fraction (5 or 6 Gy) while the other patient received a concurrent radiation sensitizer [12]. The 15 patients who received less than 3 Gy fractions did not develop dementia. Various authors now agree that the delayed late-radiation injury to the brain may be overstated and less relevant with respect to outcome [13]. However, as patients survive longer with their primary cancer long-term adverse outcomes of WBRT are of significant concern [14] and a number of trials have and continue to address these issues [15]. Without such prospective investigations it is difficult to discern the contributing effect that multiple systemic therapies can have on cognitive function.

WBRT is often used alone in RPA class III patients whose main alternative treatment option is best supportive care. In this setting, both overall response rate and neurologic improvement range from 50% to 60% [16, 17]. WBRT can also be used in conjunction with local treatment (surgery or stereotactic radiosurgery) in RPA class I/II patients whose alternative is local treatment alone or local treatment combined with systemic treatment.

### 2.3. Surgery Alone or in Combination with WBRT

Surgical excision of cerebral metastases is often used in patients with RPA class I/II, harbouring single lesions, and minimal or controlled systemic disease [18]. The goals of surgery include establishment of a diagnosis, LC in noneloquent locations, and rapid relief of symptoms (e.g., mass effect, hemorrhage, hydrocephalus). Currently, surgery involves intraoperative image guidance, microsurgical techniques, perioperative neurologic monitoring, and awake craniotomy with cortical mapping. These and other refinements [19, 20] have resulted in significant reduction in postoperative complications which can include infection, neurologic deficits (including potential impairments of cognition, speech, movement, sensation, vision, hearing, and coordination), cerebral hemorrhage, and/or infarction, the incidence of which have been reduced to under 5% [21, 22]. Postoperative mortality has been reduced to less than 1% [23]. Unlike infiltrating primary brain tumours, BMs are usually well demarcated from surrounding brain parenchyma. This increases the ability to achieve a gross total resection with a minimum of morbidity, especially new neurological deficits.

A classical and much quoted randomized controlled trial (RCT) by Patchell et al. [24] investigated the value of adding surgery to WBRT. They demonstrated that resection of a single BM leads to improved overall survival (OS; driven by increased LC), QOL, and LC compared to WBRT alone [24]. Similar results were demonstrated in another RCT [25]. However, only a quarter to a third of BM patients have a single lesion [26, 27] and of these nearly half are not suitable for surgery (inaccessibility of the tumor, extensive systemic disease, unfit for surgery, etc.). Thus, only 15% of all BM patients are surgical candidates. Furthermore, surgical resection does not eradicate microscopic disease in the operative bed nor does it address disease elsewhere in the brain. A follow-up RCT led by Patchell showed that the addition of adjuvant WBRT following surgical resection reduced recurrence at the surgical site compared to surgery alone [28]. Moreover, adjuvant WBRT following surgery prevented subsequent development of distant BMs and reduced neurological death when compared to surgery alone. OS and QOL were reported as not affected [28] raising doubt as to the value of WBRT in preserving QOL and functional independence.

### 2.4. Stereotactic Radiosurgery Alone or in Combination with WBRT

Stereotactic radiosurgery (SRS) is a technique whereby multiple convergent beams deliver high-energy X-rays, gamma rays, or protons to a discrete radiographically defined target [29]. Due to the rapid fall-off of dose outside the target volume, the radiation dose delivered to the normal brain tissue distant from the tumour is clinically insignificant. The advantages of SRS are the relative ease of tolerability for patients and the ability to treat deep-seated, surgically inaccessible lesions as well as those traditionally considered radioresistant such as melanoma [30] and renal cell carcinoma [31]. A number of SRS devices are in clinical use, including the Gamma Knife (GK, Elekta AB, Stockholm) dedicated linear accelerators such as the CyberKnife (Accuray, Sunnyvale, CA; mounted on a robotic arm) or the Novalis Tx (BrainLAB/Varian Medical Systems, Palo Alto, CA/Munich, Germany; multi-leaf collimator providing beam shaping) as well are various modified conventional linear accelerators. Radiosurgery has revolutionized the treatment of brain metastases since its first use in North America in the late 1980s [32] and, after more than 30 years of experience [33], has established itself as a valid therapeutic option providing a high degree of LC for small (≤3-up to 4 cm) metastases [34]. 

The ability of upfront SRS to improve survival has been demonstrated prospectively relatively recently [35–38]. A literature review by the American Society for Therapeutic Radiology and Oncology (ASTRO) summarized the available data according to the level of evidence [35]. Based on Level I–III evidence, for selected patients with single, small (up to 4 cm) brain metastases (up to 3 lesions and 4 in one randomized trial), the addition of SRS boost to WBRT improves brain control as compared with WBRT alone. There are two prospective randomized studies that have been published in extenso on the subject [36, 38]. The strongest evidence comes from the Radiation Therapy Oncology Group (RTOG) 95–08 trial [36], which randomized 164 patients to WBRT and SRS boost versus 167 patients to WBRT alone. Patients with one to three newly diagnosed brain metastases were included. The brain metastases could be a maximum diameter of 4 cm for the largest lesion and the additional lesions could not exceed 3 cm. For these three randomized trials, local brain control at 1 year ranged from 82% to 92% in the SRS boost arm versus 0%–71% in the whole-brain alone arm. 

Treatment with radiosurgery alone appears to result in the same overall survival as the combination of SRS and WBRT. However, local and distant brain control is significantly poorer with omission of upfront WBRT (Level I–III evidence) [36, 39]. Despite this, there is a recent trend toward withholding adjuvant WBRT from initial treatment and deferring it until other treatments have failed. The concerns are long-term neurotoxicity (especially in longer surviving patients), the availability of effective salvage treatments, and the fact that adjuvant WBRT does not translate into a prolonged OS or have an impact on preservation of performance status or functional independence [14, 40]. It seems that in patients with a limited number of brain metastases (one to three metastases), who are initially treated with either radiosurgery or surgery, WBRT can be withheld if serial imaging for followup is performed [15]. There remains a paradox that patients without active systemic disease and good performance status may be both more likely to benefit from WBRT and more likely to be harmed by its late toxicities. Patients with a poor performance status or active systemic disease may either die before developing new metastases or reseed their brain from uncontrolled extra-cranial tumors.

### 2.5. Multiple Metastases

Some of the nihilism that surrounded the management of oligometastases in the past still persists with multiple metastases patients. This is likely due to the perceived poor outcomes despite available treatments and the high incidence of concurrent, active systemic disease. There is a subset of patients, however, who have treated or controlled systemic disease and have maintained good neurologic function primarily due to small tumor size. Traditionally, fractionated WBRT to a dose of approximately 30 Gy has been administered. Surgical resection has been offered rarely to these patients, because the morbidity of resection in multiple brain locations was believed to be excessive, and the risk for developing additional tumors perceived to be high. Stereotactic radiosurgery potentially provides answers to both of the aforementioned problems.

Radiosurgery can be performed in most brain locations, irrespective of regional brain function. Accordingly, radiosurgery can be used to treat multiple metastases in one setting [41–43]. In a retrospective study of 323 patients, Chang et al. analyzed the efficacy of radiosurgery in treating patients with various numbers of brain metastases [42]. When assessing patient survival and progression-free survival times as a function of the number of BMs, they reported no statistical difference between survival times after radiosurgery. Although remote disease progression was more frequent in patients with >15 BMs, there was no statistical difference in local control rates. These findings identified SRS as a treatment option for local control of metastatic lesions and raised the notion that SRS might offer improved survival in patients with multiple metastatic brain lesions. In a separate retrospective study, Bhatnagar et al. devised a recursive partitioning analysis of 205 patients with four or more BM who were treated with SRS in one setting [41]. SRS was used alone or in conjunction with WBRT, or after failure of WBRT. With a median marginal radiosurgery dose of 16 Gy and median total treatment volume of 6.8 cc, they identified two distinct cohorts of patients. Patients with a total treatment volume of <7 cc and <7 brain metastases (4–6) were found to have extended survival following SRS. Those patients with >7 cc and >7 BMs had a significantly poorer survival following SRS. These studies confirmed a role for SRS in the treatment of patients with multiple BMs, and identified a subgroup of patients with improved survival following SRS. Like conventional surgery, however, SRS is a focal treatment and its role remains limited by the risk of development of further tumors outside the initial irradiation volumes.

## 3. Emerging Trends in Treatment of BM

With the complex needs of patients with brain metastases, therapeutic options evolve based on clinical judgement and case series. Many strategies have yet to be supported by prospective RCT's. The authors believe that two emerging treatment paradigms will soon integrate the mainstream and thus warrant further clinical studies.

### 3.1. Radiotherapy to the Surgical Bed

The postoperative delivery of WBRT for patients with BM aims to sterilize residual disease in the tumor bed as well as other sites of occult disease in the brain. Reviewing the RCT data from Patchell et al., in the absence of WBRT, 46% of patients with an MRI confirmed complete resection had a recurrence in the original site at a median of 27 weeks. The addition of high-dose WBRT (50.4 Gy) decreased this recurrence to 10% with a time to recurrence of ≥52 weeks [28]. This series, as most others, did not censure patients for LC at the time of last imaging leading to failure rate underestimations. In addition, few physicians use such high doses of WBRT in clinical practice either because of resource limitations or for fear of neurocognitive effects. Finally, complete resection is not always confirmed by immediate post-operative MR imaging. With these caveats, the actual 1-2 year local recurrence rate is likely often higher in clinical practice.

Stereotactic radiosurgery to the surgical bed in addition to or without WBRT is an emerging trend in the treatment of brain metastases. A retrospective review carried out at the McGill University Health Center, Montreal, Canada (MUHC) of patients treated with surgery followed by WBRT revealed an actuarial rate of local recurrence of 67% at 2 years [44]. A retrospective series from Rades et al. [45, 46] compared 2 groups of patients with RPA class I to II disease with 1-2 resectable brain metastases: resection plus WBRT and same treatment plus a fractionated boost to the tumor bed (5 fractions of 3 Gy or 5 fractions of 2 Gy each). In this series, the boost improved both LC (2-year LC after complete resection 88% versus 32%, *P* < 0.001) and overall survival (1-year OS 66% versus 41%, *P* < 0.001). In a 5-year experience at the MUHC using WBRT plus a tumor bed SRS boost of 10 Gy in 44 patients, the actuarial 2-year LC was 91%. The median survival was 17 months, and with a median followup of 10 months, only 11% developed new metastases [47–49]. Without the SRS boost, the 1- and 2-year actuarial LC rates were 52% and 33%, respectively, statistically worse than with tumor bed SRS (*P* < 0.001) and in keeping with the data of Rades et al. [45, 46].

Some institutions advocate radiosurgery alone as adjuvant treatment. Three recent retrospective studies have described the use of SRS or fractionated stereotactic radiotherapy following surgery without WBRT. Soltys et al. reported a 1-year actuarial LC rate of 79% with no deaths secondary to neurologic causes in a series of 72 patients treated at Stanford University [50]. In a series of 40 patients, Mathieu et al. reported a crude LC of 73% (2-year actuarial LC of 60%) [51]. One-fifth of patients had only a partial resection. Do et al. demonstrated that an actuarial 1-year LC rate was 82% in 30 patients, 19 (63%) of which developed recurrences in new intracranial sites [52]. WBRT was administered only as salvage treatment in 14 of the 30 patients. 

The concern with tumor bed SRS alone is that SRS is limited by the size of the tumor cavity and prone to geographic miss. Some groups have advocated the inclusion of a 2 mm margin around the tumor cavity to improve LC [50]. As previously discussed, WBRT has a proven effect in reducing the occurrence of new brain metastases. In Patchell's study, WBRT decreased the occurrence of new metastases from 37 to 14% [28]. Aoyama et al. demonstrated a reduction from 64 to 42% with no difference in toxicity [39]. In addition, the above studies looking at SRS ± WBRT demonstrated greater local control rates with intact brain metastases. It seems reasonable to expect that in a similar fashion, the combination of SRS and WBRT would show improved local control at the surgical bed. Moreover, WBRT eliminates any risk of complete marginal miss from difficulties in defining a proper post-operative target. The use of a reduced dose of SRS opens this option to patients with large tumors and lesions approximating critical structures. These patients can thus be treated without compromising dose intensity.

In summary, post-operative intracranial failure most commonly occurs in the tumor bed, despite the use of adjuvant WBRT. Although evidence suggests that postoperative SRS provides increased LC, a prospective trial comparing this strategy to WBRT alone has not been carried out and is the subject of a current National Cancer Institute trial (NCT00003320). If the number of published series can be used as an indicator, tumor bed SRS alone is being used more and more commonly. If this strategy can provide equivalent LC to WBRT with less neuro-cognitive toxicity, its adoption will grow rapidly.

### 3.2. Salvage Following Initial Treatment Failure

Advances in chemotherapeutic agents for systemic cancer have improved patient survival and overall prognosis. As the number of patients with prolonged survival rises, and the use of upfront WBRT decreases, an increase in a subgroup of patients with BM who have failed initial treatment and developed recurrence is likely. Although previously neglected, this growing subgroup merits specific studies to determine the optimum treatment strategy to extend survival and minimize morbidity. 

At our institution, over a third of the eligible patient population undergo upfront SRS alone. The therapeutic strategies offered to these patients continue to evolve but finding Level 1 evidence to guide physicians remains challenging, as studies in this area are very limited.

Brain relapses comprise not only lesions that have progressed locally following initial treatment but also new lesions that have developed at a distance or a combination of the two. In this context, surgical resection is often not appropriate and not indicated as more than two-thirds of patients have multiple brain metastases and large tumors are scarce as closer MRI followup allows early detection of new metastases. Current chemotherapy regimens or small molecule inhibitors used for targeted therapy of systemic cancer are typically not effective for BM as the blood-brain barrier limits bioavailability in the tumour tissue [53]. Nonsurgical salvage treatment options include WBRT for WBRT-naïve patients, repeat WBRT, salvage SRS, or a combination of radiation modalities.

#### 3.2.1. Salvage WBRT

Although Salvage WBRT alone is a widely accepted traditional approach at most institutions, there are no studies that evaluate its use in patients whose initial management did not include WBRT, in other words salvage WBRT after failure of initial treatment with SRS. Conversely, the use of repeat WBRT in patients who have failed previous treatment with WBRT has been reported but its use remains controversial and doubts persist as to the therapeutic ratio [54–57]. Largely retrospective data show improvement in neurologic function in 31–42% of patients, median duration of response of 2.5 months, and median survival of 4-5 months following reirradiation with 20–25 Gy over multiple fractions. The strongest predictors of a favourable outcome following re-irradiation were a good performance status and a good response to initial treatment. Late complications are largely unreported but proponents argue that these need to be balanced by the likely occurrence of neurological deterioration if lesions are left untreated [56].

#### 3.2.2. Salvage Radiosurgery

Salvage radiosurgery as defined by the use of SRS to treat brain relapses is being offered on an individual basis and is in many cases accepted as sole treatment. Indeed, this indication was at the very origin of the use of SRS for BM [58]. Although its role at the time of progressive brain metastases previously treated with either whole-brain radiotherapy or SRS alone has not been fully elucidated, there is evidence to suggest that it provides good LC thus extending BDFS [59]. However, there are theoretical concerns of higher rates of radiation necrosis associated with repeat SRS at local failure with the consequent increased risk of neurological deficits.

A number of retrospective case series exist analyzing salvage SRS in patients whose initial management was WBRT along [59–62]. These studies date back to the early 1990s where Loeffler et al. showed 100% LC in 21 lesions [59]. In one of the larger series of the subsequent decade, Noël et al. [62] reported 1- and 2-year LC rates of 91 and 84% with a median survival of 7.8 months in 54 patients whose median interval between the end of WBRT and salvage SRS was 9 months. One- and 2-year new brain event-free survival rates were 65% and 57%, respectively. Although 24% of patients developed new metastases, none of the patients died from cerebral cause, and only 5% of the treated lesions recurred with 28% of patients being still alive at 2 years. Only 7% of patients developed complications including mild headaches and alopecia. In a larger more recent study, Chao et al. showed an overall survival of 9.9 months following salvage SRS in 111 patients [60]. LC was 68 and 59% at 1 and 2 years, respectively, with 25 and 31% of patients developing local and distant recurrence, respectively. Only 3.6 % of patients developed complications (radiation necrosis in 2 patients, seizures in 1, and severe fatigue in 1). One prospective study showed a median survival of 6 months from salvage SRS with LC of 19 treated lesions [61]. Time to progression was not reported and 75% of the lesions eventually recurred. Although predictive factors were not consistent in the literature, the interval between WBRT and SRS seem to predict overall survival.

A few studies examine the use of salvage SRS after initial treatment with SRS [63–66]. Two of these studies present survival data from the date of salvage SRS [63, 64]. Kwon et al. presented a series of 43 patients who underwent salvage SRS in which median survival from the time of SRS to recurrent/progressive disease was 32 weeks and the LC rate of retreated lesions at 6 months was 91% [64]. Multivariate analysis revealed that RPA class was the only predictor for overall survival. In the case series by Chen et al., of 45 patients, median survival from the time of SRS for recurrent brain metastases was 28 weeks [63]. The 1-year freedom from progression rate was 94%. Both authors noted that repeated SRS may extend survival at least as long as the first SRS intervention had. Multivariate regression analysis failed to reveal factors predictive of survival. Unfortunately, these studies are retrospective and the reported results are subject to selection bias. No comparison is made with other competing strategies, making the evidence class II and III [67]. In addition, predictive factors are inconsistent. Nevertheless, they provide compelling support for repeat or salvage SRS being effective in treating locally progressive or new lesions after initial SRS. Arguably, this strategy should be considered in favourable RPA class patients in order to minimize morbidity, maximize patient quality of life and perception of disease burden [60], and to reduce cost [68–71]. Prospective trials are needed to determine the clinical value and compare SRS and WBRT salvage modalities at the time of local or distant failure.

The goal of brain metastases management is to minimize morbidity and mortality, improve patient quality of life, and reduce associated treatment costs. In an era of healthcare budget constraints, it is imperative that clinicians adopt fiscally responsible standards to guide their practice. Several reports have examined the treatment outcomes and cost-effectiveness of current management paradigms. In a retrospective study, Metha and colleagues assessed the survival and quality of life outcome data for patients with solitary brain metastasis, who were randomized to receive WBRT, surgery + WBRT, or radiosurgery alone [69]. When comparing the relative cost ratios of surgery and radiosurgery, and analyzing the cost-effectiveness (cost per year of median survival) of each modality, radiosurgery yielded greater survival and functional independence versus surgery or WBRT alone. Rutigliano et al. reported similar findings in a separate retrospective study [70]. More recently, Lee et al. examined the outcomes and cost-effectiveness of treating 156 patients with multiple brain metastases, randomized to receive radiosurgery or WBRT [68]. The follow-up time in this study was 3.3 years. The mortality rate for radiosurgery-treated patients, with multiple brain metastases (2–5 lesions) and a good initial KPS score, was found to be significantly better. Radiosurgery also resulted in better posttreatment KPS scores, improved quality of life, and higher cost-effectiveness when compared to WBRT. These findings highlight the need for prospective clinical trials to comprehensively study the clinical and economic efficacy of various treatment modalities, especially in a time of increasing healthcare costs and significant budget constraints.

## 4. Conclusions

Over the last three decades a major shift in the philosophy guiding the treatment of patients with brain metastases has occurred. The traditional nihilistic expectation of rapid neurological decline and inevitable neurological demise is no longer acceptable, and the focus has moved from palliation to achieving sustained brain disease control, systemic disease control, and preserved neurological function. Thus, with a variety of aggressive treatment options available to them, patients can now benefit not only from longer overall survival but also extended brain disease-free survival and improved quality of life. These parameters and others including the prolongation of functional independent status, reduction in burden of focal neurological deficits, neurocognitive preservation, and freedom from seizures have now become established treatment goals and have taken over overall survival as primary endpoints in major brain metastases trials.

With current management strategies, patients with limited brain metastases are often more likely to succumb from their systemic disease than from their brain tumour(s) [28]. Continual progress in systemic cancer treatments has led to a steady increase in patient survival and overall prognosis. Paradoxically, as control of systemic disease improves, the occurrence of brain metastases and its associated neurological morbidity and mortality will increase. 

The management of brain metastases has seen a paradigm shift from palliation toward aggressive intervention to achieve control of brain disease. Following surgical resection, adjuvant WBRT has been shown to decrease death due to neurological causes. However, more recent data has highlighted the potential benefit of more aggressive local control measures involving surgical resection and SRS in addition to WBRT. Radiosurgery alone, surgical resection with adjuvant SRS, and advanced chemotherapeutic agents have been increasingly used amidst concerns of the potential neurotoxicity of WBRT. 

Following surgery, intracranial failure most commonly occurs in the tumor bed, despite WBRT. Postoperative SRS boost to the tumor cavity may provide acceptable local control without many of the toxicities of WBRT. The efficacy of this strategy remains to be tested in a comparative prospective trial. Optimum management strategies at the time of failure remain unclear and include salvage WBRT, salvage SRSs or a combination of the two. 

In summary, although much progress has been made in the last 3 decades, numerous challenges lay ahead in establishing evidence-based guidelines in this challenging group of patients. The field of neuro-oncology is witnessing an exciting evolution in management of brain metastases. The future holds great promise and opportunity to carry out focused clinical investigations to demonstrate efficacy of our treatment paradigms. Proposed management goals will not only aim to increase overall survival and brain disease-free survival, but also to improve quality of life and prolong functional independent status whilst minimizing neurological deficit.

## Figures and Tables

**Table 1:** RTOG (RPA) recursive partitioning analysis [6] (modified by age).

Class	Variables
Class I	KPS ≥ 70, controlled primary tumor, metastases to brain only, age < 76 years

Class II	KPS ≥ 70, but uncontrolled primary tumor, age < 76 years
	KPS ≥ 70, primary controlled, but metastases to brain and other sites, age < 76 years

Class III	KPS < 70
